# Nanoscale Noncollinear Spin Textures in Thin Films of a *D*_2d_ Heusler Compound

**DOI:** 10.1002/adma.202101323

**Published:** 2021-07-03

**Authors:** Ankit K. Sharma, Jagannath Jena, Kumari Gaurav Rana, Anastasios Markou, Holger L. Meyerheim, Katayoon Mohseni, Abhay K. Srivastava, Ilya Kostanoskiy, Claudia Felser, Stuart S. P. Parkin

**Affiliations:** ^1^ Max Planck Institute of Microstructure Physics Weinberg 2 06120 Halle Germany; ^2^ Max Planck Institute for Chemical Physics of Solids Nöthnitzer Str. 40 01187 Dresden Germany

**Keywords:** Heusler thin films, Mn _2_RhSn, noncollinear spin textures, skyrmions, spintronics

## Abstract

Magnetic nano‐objects, namely antiskyrmions and Bloch skyrmions, have been found to coexist in single‐crystalline lamellae formed from bulk crystals of inverse tetragonal Heusler compounds with *D*_2d_ symmetry. Here evidence is shown for magnetic nano‐objects in epitaxial thin films of Mn_2_RhSn formed by magnetron sputtering. These nano‐objects exhibit a wide range of sizes with stability with respect to magnetic field and temperature that is similar to single‐crystalline lamellae. However, the nano‐objects do not form well‐defined arrays, nor is any evidence found for helical spin textures. This is speculated to likely be a consequence of the poorer homogeneity of chemical ordering in the thin films. However, evidence is found for elliptically distorted nano‐objects along perpendicular crystallographic directions within the epitaxial films, which is consistent with elliptical Bloch skyrmions observed in single‐crystalline lamellae. Thus, these measurements provide strong evidence for the formation of noncollinear spin textures in thin films of Mn_2_RhSn. Using these films, it is shown that individual nano‐objects can be deleted using a local magnetic field from a magnetic tip and collections of nano‐objects can be similarly written. These observations suggest a path toward the use of these objects in thin films with *D*_2d_ symmetry as magnetic memory elements.

## Graphical abstract

Noncollinear magnetic nano‐objects are observed in epitaxial thin films of the *D*_2d_ Heusler compound Mn_2_RhSn using magnetic force microscopy. The nano‐objects are stabilized over a wide temperature‐field region and can be deleted or created by local magnetic fields, thereby paving a path toward the realization of skyrmionic devices based on *D*_2d_ Heusler thin films.

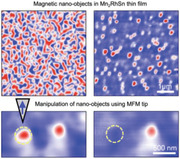

## 1 Introduction

Recently, magnetic skyrmions have received much attention. These topologically protected, noncollinear magnetic spin textured nano‐objects^[^ ^1^, ^2^, ^3^, ^4^, ^5^, ^6^, ^7^ ^]^ are stabilized in magnetic compounds with broken inversion symmetry, and are the result of a competition between a chiral Dzyaloshinskii–Moriya interaction (DMI)^[^ ^8^, ^9^ ^]^ and a ferromagnetic exchange interaction. Perhaps the most extensively studied spin textures are, first, Bloch‐like skyrmions that have been observed in noncentrosymmetric B20 compounds, both in single crystals^[^ ^5^ ^]^ and in epitaxial films,^[^ ^10^ ^]^ and, second, Néel‐like skyrmions in thin‐film heterostructures formed from ultrathin ferromagnetic layers and a heavy metal layer.^[^ ^6^, ^11^ ^]^ The former relies on a volumetric and the latter an interface derived DMI.

In recent studies, the family of inverse tetragonal Mn_2_YZ‐based Heusler compounds has been shown to sustain magnetic antiskyrmions,^[^ ^12^, ^13^, ^14^ ^]^ another type of noncollinear spin texture that exhibits distinct topological characteristics, and, in addition, elliptical Bloch skyrmions.^[^ ^15^ ^]^ These textures are a result of the underlying *D*_2d_ crystal symmetry that necessarily gives rise to an anisotropic DMI. This DMI also leads to an enhanced stability of antiskyrmions with respect to field and temperature and, the extreme tunability of their size by simply varying the thickness of the lamella in which exist.^[^ ^16^, ^17^ ^]^ The latter is a result of dipole–dipole interactions that is important in compounds with low symmetry, such as *D*_2d_ and also accounts for the possibility of elliptical Bloch skyrmions in the same material system.^[^ ^15^, ^18^, ^19^ ^]^

To date, all of these observations of noncollinear spin textures in *D*_2d_ systems have been made using lamella extracted from bulk crystals. However, their observation in thin‐films is desired for technological applications, such as Racetrack memory devices.^[^ ^20^ ^]^ It remains challenging to make thin films of tetragonal Heusler compounds. In single crystals, high degrees of chemical ordering are achieved by very high temperature annealing processes, but this is not possible in thin‐films. This is especially true for the complex thin‐film hetero‐structures that are needed for technological applications, where even modest annealing temperatures leads to intermixing of the constituent layers.

Skyrmions can be observed in real‐space by various direct imaging techniques.^[^ ^4^, ^21^, ^22^, ^23^, ^24^, ^25^, ^26^ ^]^ Here, we use magnetic force microscopy (MFM) imaging to investigate magnetic textures in thin films of [001]‐oriented Mn_2_RhSn. We observe magnetic nano‐objects over a wide range of temperature (from 2 to 280 K) and magnetic field. We compare these textures with those that we find, via Lorentz transmission electron microscopy (LTEM), in single crystals of the same material. Elliptical and round shaped isolated objects are formed with varying sizes that depend upon the field and temperature. Additionally, we also demonstrate the creation and annihilation of these objects with the help of local magnetic field gradients generated by an MFM tip.

## 2 Results and Discussion

**Figure 1:**
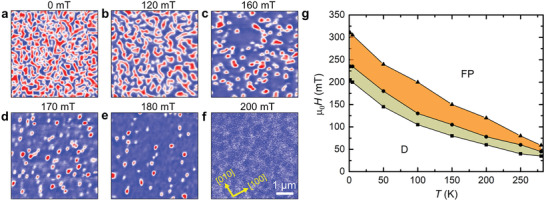
Evolution of nanoscale magnetic textures in a 35 nm‐thick Mn_2_RhSn film imaged by MFM. a) MFM image at 100 K and zero magnetic field. b–f) Evolution of the nanoscale objects as the magnetic field is increased from 120 to 180 mT and finally to the field polarized state at 200 mT. The blue and red colors correspond to up and down domains respectively. g) Magnetic field versus temperature phase diagram. The khaki‐colored area shows a mixed phase of labyrinth and isolated nano‐objects, and the orange area a single phase of nano‐objects. D and FP correspond to labyrinth domains and field polarized state, respectively. All MFM images are at the same scale: a scale bar is given in (f).

The studies here were carried out on high‐quality epitaxial thin films of Mn_2_RhSn that were reported previously^[^ ^27^ ^]^ (see the Experimental Section). The films were prepared on MgO (001) substrates using DC magnetron sputtering. The observation of a topological Hall effect in these films suggests the presence of noncollinear spin textures.^[^ ^27^, ^28^ ^]^ Here, we have carried out variable temperature magnetic force microscopy imaging of several Mn_2_RhSn films. We focus on a 35 nm‐thick film. The magnetic tip in the MFM is first magnetized in a direction perpendicular to the film surface. The sample is cooled down from 325 K, above the Curie temperature *T*_c_ ≈294 K, to low temperatures in zero magnetic field. Typical MFM results taken at 100 K as a function of increasing magnetic field are summarized in **Figure** **1**. The initial state after cooling in zero magnetic field is shown in Figure 1a. A labyrinth magnetic domain structure is observed in which blue and red colors correspond to regions with magnetization pointing up and down into the plane of the film, respectively. As the magnetic field is increased from zero, the extent of the blue regions increases and, correspondingly, the total extent of the red regions decreases. The labyrinth pattern is retained for small magnetic fields, as shown in Figure 1b at 120 mT, but then gradually transforms into an array of irregularly shaped isolated objects at higher fields, as shown in Figure 1c,d. These discrete nano‐objects, as discussed later, appear to be elliptically shaped, but at even higher magnetic fields (Figure 1e) the objects become circularly shaped. A saturated magnetic state is achieved at ≈200 mT. The size of the nano‐objects varies from ≈190–225 nm depending on the temperature (see Figure S1 in the Supporting Information).

Similar measurements were performed at various temperatures. The labyrinth domain phase was observed at all temperatures from 2 to 280 K, but the range of magnetic field over which this structure was found increased with decreasing temperature. The range of field for which isolated objects was found had a weaker temperature dependence, decreasing only as the Curie temperature is approached. These field regions are plotted in the field–temperature phase diagram in Figure 1g.

**Figure 2:**
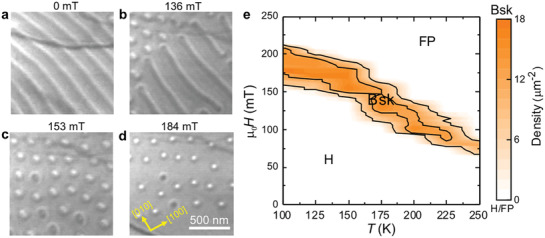
Observation of Bloch skyrmions in a 150 nm‐thick Mn_2_Rh_0.95_Ir_0.05_Sn single‐crystal lamella imaged by LTEM. a) Helical phase at zero field and 150 K. b–d) Magnetic field evolution of Bloch skyrmions. The circles with white and black interiors correspond to Bloch skyrmions with opposite chiralities. All images are at the same scale: a scale bar is given in (d). e) Magnetic field versus temperature phase diagram. Bloch skyrmions are nucleated by applying a temporary tilting angle of 32° along the [100] crystallographic direction. H, Bsk, and FP correspond to helical phase, Bloch skyrmions and field polarized state, respectively.

Results on the Mn_2_RhSn thin films were compared with results on a thin ≈150 nm‐thick [001] zone‐axis oriented lamella that was formed from a single‐crystalline grain of a bulk polycrystalline Mn_2_Rh_0.95_Ir_0.05_Sn sample. The lamella was formed using focused ion milling techniques, as discussed, for example, in ref. ^[^ ^14^ ^]^. This specimen was examined using LTEM. The field dependent evolution of the spin texture at 150 K is shown in **Figure** **2**a–d. LTEM is sensitive to the in‐plane magnetization component, as distinct from MFM that is sensitive to the out‐of‐plane magnetization component. The initial image taken after zero field cooling is quite different from that seen in the thin‐film sample discussed above. Rather than a labyrinth structure, an alternating series of black and white regions is observed that is consistent with a helical magnetic structure. This helix has a propagation direction that is oriented approximately along the in‐plane [100] direction. As the field is increased the magnitude of the helical period increases significantly and the helical phase is gradually replaced by arrays of elliptically shaped objects. The shape of the nano‐objects becomes more circular at higher magnetic fields, for example, at 184 mT (Figure 2d). From the LTEM studies these nano‐objects can be identified as Bloch‐like skyrmions since they can be observed at a zero‐tilt angle, θ, of the lamella with respect to the direction of the transmitted electron beam. Note that Néel‐like skyrmions display no contrast under these conditions. The nano‐objects observed in Figure 2c display an LTEM contrast with either white or black interiors. These correspond to Bloch skyrmions with opposite chiralities^[^ ^15^ ^]^ that were first observed in Mn_1.4_Pt_0.9_Pd_0.1_Sn. In an inverse tetragonal Heusler compound the major axes of such elliptical objects are tied to the crystallographic directions [100] and [010]. However, here for Mn_2_Rh_0.95_Ir_0.05_Sn, these objects are not strictly tied to a particular crystallographic direction. We speculate that this difference arises from the lower magnetization of Mn_2_Rh_0.95_Ir_0.05_Sn and the consequent lesser importance of dipole–dipole interactions that stabilize the Bloch skyrmions. We note that anti‐skyrmions have previously been observed in Mn_2_RhSn in a single‐crystalline lamella but under different field–temperature history protocols.^[^ ^14^ ^]^ The LTEM results are summarized in the field–temperature phase diagram shown in Figure 2e.

In the single‐crystalline, 150 nm‐thick lamella, the size of the nano‐objects is ≈130–200 nm, depending on temperature (see Figure S1, Supporting Information). This is comparable to the size of the nano‐objects in the film discussed above that was 35 nm thick. However, a very strong increase in size of the magnetic nano‐objects in Mn_1.4_PtSn has been reported in single‐crystalline lamella as the lamella thickness along [001] was increased. This was reported to be due to the influence of magnetic dipole–dipole coupling, that can be important in compounds with *D*_2d_ symmetry.^[^ ^16^ ^]^ To further explore the role of dipole coupling in thin films of Mn_2_RhSn, we have carried out MFM imaging of spin textures in a [001]‐oriented film that is 110 nm thick. As shown in Figure S2 (Supporting Information), the spin textures are similar to those we find in the thinner 35 nm‐thick film, but the size of the nano‐objects is only slightly larger, varying from ≈245 to 280 nm. Thus, we conclude that, just as in the single‐crystal lamella mentioned above, with regard to the orientation of the Bloch skyrmions, dipole–dipole coupling plays a less important role in Mn_2_RhSn than in Mn_1.4_PtSn.

A comparison of the phase diagrams for the lamella and the 35 nm‐thick thin film of Mn_2_RhSn (Figures 1g and 2e) show that the field–temperature stability windows for the observation of discrete nano‐objects is similar in both cases. Moreover, in neither case are well‐ordered arrays of these objects found, in distinct contrast to Mn_1.4_Pt_0.9_Pd_0.1_Sn, where hexagonal arrays of both antiskyrmions or Bloch skyrmions are typically observed.^[^ ^15^ ^]^ After the sample's magnetization is saturated in a large magnetic field, when the field is swept from saturation to small negative fields, no nano‐objects are found either in the thin film or the lamella but, rather, the labyrinth state in the one case, and the helical phase in the other case is found. (Note that Bloch skyrmions can be stabilized in the lamella when the field is decreased before the helical phase is formed, by using an additional in‐plane field component that is applied by tilting the sample in the transmission electron microscope, as shown in Figure S3 (Supporting Information). The field evolution of magnetic textures in the negative field direction also produces similar nano‐objects in MFM (for thin‐film) and LTEM (for single‐crystalline lamella) following the same procedures used as in Figures 1 and 2, respectively (see Figures S4 and S5, Supporting Information). In none of these experiments was any evidence found for pinning of the magnetization textures to specific sites in the film or lamella (as shown in Figures S4 and S5, Supporting Information).

**Figure 3:**
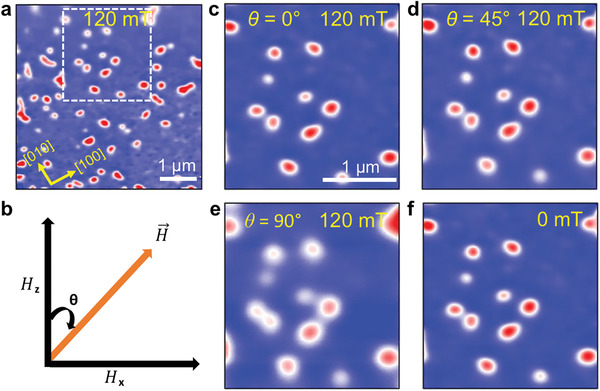
Robust stability of nano‐objects in a 35 nm‐thick Mn_2_RhSn film in a vector magnetic field. a) Isolated nano‐objects at 120 mT and 200 K. b) Illustration of field H applied at an angle θ, where *H_z_* (θ = 0°) and *H_x_* (θ = 90°) are out‐of‐plane and in‐plane fields applied to the film. The area marked by the white dashed square is shown enlarged in (c)–(f). c) Portion of (a). d–f) MFM images in the presence of *H* = 120 mT, after the angle θ has been changed from 0° to 45° and 90°, in (d) and (e) respectively, and after the field has been reduced to zero in (f).

To further investigate the robustness of the nano‐objects observed in thin films of Mn_2_RhSn, we performed MFM measurements for the 35 nm‐thick film in the presence of a vector magnetic field. First, following the aforementioned procedure of Figure 1, isolated nano‐objects were stabilized at 120 mT and 200 K, as shown in **Figure** **3**a. Then, the field is rotated from θ = 0°, the normal to the sample plane, toward the sample plane. The nano‐objects are stable to this procedure, as shown in Figure 3c–e, as θ is increased from 0° to 45° and then to 90°. When the magnitude of the field is then reduced to zero, the nano‐objects remain, as shown in Figure 3f. This contrasts with the case when the perpendicular field magnitude is reduced to zero without any tilt when, as discussed earlier, the labyrinth state is formed. These results emphasize the sensitivity of the magnetization texture in Mn_2_RhSn films to the field history protocol.

**Figure 4:**
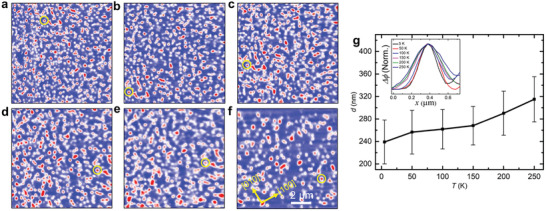
Temperature dependence of nanoscale objects in a 35 nm‐thick Mn_2_RhSn thin. a–f) MFM images in 10 mT at a temperature of 5, 50, 100, 150, 200, and 250 K, respectively. g) Size dependence of nano‐objects with temperature. The error bars represent the standard deviation of the nano‐object size. The inset shows the line profiles of the same nano‐object that is highlighted by the yellow circles in (a)–(f). All images are at the same scale: a scale bar is given in (f).

To reinforce the sensitivity of the magnetization state of the 35 nm‐thick thin‐film sample to the temperature‐field history, a field cooling procedure was carried out from above the Curie temperature to low temperatures in the presence of an out‐of‐plane magnetic field. Here a field of 10 mT was applied as the sample was cooled from 325 to 5 K. As shown in **Figure** **4**a, a collection of nano‐objects is now found at 5 K, in sharp contrast to the labyrinth state that is observed when cooling without any magnetic field (see Figure S6, Supporting Information). As the temperature is subsequently increased, the image remains qualitatively the same up to a temperature of ≈150 K. Above this temperature, however, the size of the nano‐objects gradually increases. Individual nano‐objects could be followed as a function of temperature, as shown in Figure 4. The average size of the objects is summarized in Figure 4g as a function of temperature. The line‐profiles of a nano‐object highlighted by the yellow circles in Figure 4a–f are plotted in the inset of Figure 4g. The size was calculated using the method described,^[^ ^17^ ^]^ which agrees well with the size determined from the line profiles of the nano‐object. These low‐field stabilized nano‐objects are metastable and, therefore, have a larger size than the field‐driven nano‐objects as shown in Figure 1. The density of the nano‐objects was found to decrease with increasing temperature. Similar experiments were carried out for the lamella, as shown in Figure S7 (Supporting Information).

**Figure 5:**
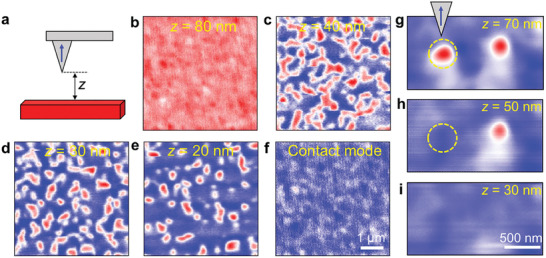
Controlled creation and annihilation of nano‐objects in a 35 nm‐thick Mn_2_RhSn thin film. a) Schematic illustration of magnetization orientations of MFM tip and sample for writing. The distance between tip and the sample is the scan height *z*. b–e) MFM images in zero field and *z* = 80, 40, 30, and 20 nm, respectively at 200 K. f) Contact‐mode image in zero field and 200 K. The blue and red colors represent up and down magnetization, respectively. Images in (b)–(f) are at the same scale: a scale bar is given in (f). g–i) MFM images taken at *z* = 70, 50, and 30 nm at 100 K under *H_z_* = 180 mT. Images in (g)–(i) are at the same scale: a scale bar is given in (i).

Finally, we explore the writing and deleting of nano‐objects in Mn_2_RhSn thin films, using the stray magnetic field from an MFM tip, in the absence of any applied field. The field generated by the tip must be equal to or larger than the nucleation field for the nano‐object. The MFM tip used for all the images discussed above was too small for this purpose and played little or no role in the MFM images presented so far. To accomplish writing of nano‐objects, a different MFM tip was used which had a much larger stray field (see the Experimental Section). The following procedure for the 35 nm‐thick film was used at 200 K. First, the magnetization of the sample and the MFM tip were set in the same direction by applying an external field of 200 mT at 250 K. Then, the sample magnetization alone was reversed by applying a field of −80 mT (the coercivity of the sample at this temperature is 70 mT, whereas the tip has a coercivity of ≈90 mT). The external field was then reduced to zero and the sample was cooled to 200 K in zero field. Finally, writing was carried out, as shown in the sequence of MFM images in **Figure** **5**a–e. These images are taken in a noncontact mode, i.e., at a given fixed tip to sample distance, *z*. First, the scan was carried out at *z* = 80 nm, where no magnetic contrast is observed (see Figure 5b). At this height, the stray field of the tip is too small to influence the sample magnetization. When *z* is reduced to below 50 nm, the sample magnetization begins to reverse with the formation of a labyrinth pattern. When *z* is decreased below 30–20 nm, the labyrinth evolves into isolated nano‐objects. (Note that nano‐objects can also be written in contact mode, as shown in Figure S8 (Supporting Information).

To delete the nano‐objects, the magnetization of the MFM tip should be aligned in the same direction as the external field. The field from the tip adds to the external field, which, thereby, can lead to the annihilation of a nano‐object. An example is shown in Figure 5g for the 35 nm‐thick sample. From the zero‐field magnetic state at 100 K, nano‐objects are first created by applying an external field of 180 mT. Two isolated nano‐objects are shown in Figure 5g. When *z* = 70 nm, the stray field from the tip is too weak to perturb the nano‐objects. When *z* is reduced to 50 nm (Figure 5h), the first nano‐object highlighted in the dashed circle is annihilated and the second nano‐object is annihilated when *z* is further reduced to 30 nm.

We suggest that the variation in sizes of the isolated magnetic objects and the absence of helical order in our thin films can be attributed to chemical inhomogeneity on the atomic scale. Although high‐resolution transmission electron microscopy on similar films shows no clear evidence for any such inhomogeneity,^[^ ^27^, ^28^ ^]^ we have carried out X‐ray diffraction experiments on the 35 nm‐thick film using a high brilliance gallium‐Jet X‐ray source. We find that the film structure is characterized by the presence of about 6 at% of vacancies at the Mn and Rh sites, while a few percent of Mn atoms occupy otherwise empty sites in the unit cell. From this analysis the film composition is derived to be Mn_2.02_ Rh_0.94_Sn_1.00_ (see Figures S9 and S10 (Supporting Information). It is a challenge to realize high levels of chemical ordering in thin films of Heusler compounds because typically this requires high temperatures, as is used, for example, in the growth of high‐quality single crystals of Heusler compounds.^[^ ^14^ ^]^ One method that has been shown to give rise to chemical ordering at temperatures as low as even room temperature for binary Heusler compounds is the chemical templating (CTL) method.^[^ ^29^ ^]^ It is of great interest to explore this method in the future and see whether it can be extended to ternary Heuslers. If one can prepare ultrathin *D*_2d_ Heusler films, the current driven motion of noncollinear nanoscale objects in such films driven by spin–orbit torques can be explored by combining these films with layers in which there is significant charge to spin conversion.^[^ ^22^, ^30^, ^31^ ^]^ To date, we have not seen any significant motion of these objects in lamellae or thin films from volume spin transfer torques generated by current passed directly through them.

## 3 Conclusion

Nanoscale spin textures are observed in thin films of [001]‐oriented Mn_2_RhSn using MFM and compared with those observed in a single‐crystalline lamella using LTEM. We find that the stability region of nano‐objects with respect to temperature and magnetic field is extensive and similar in both cases. In neither case are well ordered arrays of the magnetic nano‐objects found. However, a labyrinth domain structure is observed in the thin film, in contrast to the bulk lamella where a helical structure is found under similar field–temperature conditions. We find evidence of a small increase in size of the magnetic nano‐objects with increasing film thickness, but which is a much smaller variation than previously seen in a related Heusler compound with *D*_2d_ symmetry. This suggests that magnetostatic energy plays a less important role in Mn_2_RhSn, which we attribute to its comparatively lower magnetization. Detailed X‐ray structural studies of the thin films suggest that there is chemical inhomogeneity that may account for the absence of a helical phase and the large size variation of the magnetic nano‐objects. In films that had even greater degrees of chemical inhomogeneity no magnetic nano‐objects were seen nor was the labyrinth phase found, providing further evidence that the observation of well‐defined spin textures in thin Heusler films requires highly chemically ordered films.

Our studies suggest that the nano‐objects we have found in thin films of Mn_2_RhSn are likely Bloch skyrmions or antiskyrmions, that we observe in single‐crystalline lamella of the same material. Moreover, we have demonstrated that individual nano‐objects can be deleted or created by local magnetic fields. Our work paves the way to the realization of skyrmionic devices based on Heusler thin films.

## 4 Experimental Section

### Thin‐Film Growth

Thin films of Mn_2_RhSn were grown on MgO (001) substrates in a BESTEC UHV magnetron sputtering system using three independent Mn, Rh, and Sn sources. The films were grown by cosputtering at 400 °C and then post annealed in situ for 30 min to improve chemical ordering. To prevent oxidation, the films were capped with 3 nm‐thick TaN films that were deposited at room temperature. The film structure and stoichiometry were characterized using X‐ray diffraction (XRD), X‐ray photoelectron spectroscopy (XPS), and Rutherford backscattering spectrometry (RBS).

### XRD

The crystal structure of the Mn_2_RhSn thin films was investigated by XRD using a Ga‐Jet X‐ray source operated at 70 keV and 100 W power emitting Ga‐Kα radiation (λ = 1.3414 Å) and a six‐circle diffractometer specially designed for the study of thin films. The primary beam is incident on the sample under a constant grazing incidence angle (μ = 1°) to the surface. Integrated reflection intensities were collected by rotating the sample around its surface normal while the 2D pixel detector is kept at a constant angle to accept the scattered beam. The structure analysis was carried out by least squares refinement of calculated structure factor magnitudes to the observed ones.

### MFM

MFM measurements were carried out in a variable temperature system from Attocube (attoAFM I) equipped with a vector superconducting magnet, so that magnetic fields can be applied both in‐plane and out‐of‐plane to the sample (attoLIQUID2000). All measurements were carried out in vacuum with a large quality factor (*Q* > 30 000) of the cantilever that allows for high force sensitivity. A magnetic probe from Nanosensors (SSS‐QMFMR) with a tip radius of ≈20 nm was used for imaging the magnetic textures using a phase modulation technique in noncontact mode. MFM imaging was carried out in two steps. First, the tilt and misalignment of the sample was corrected and topography was acquired. Then, to measure magnetic forces, the cantilever was retracted from the sample surface and scanned at a fixed lift height (50–60 nm). For writing and deleting the nano‐objects, magnetic probes from Nanoworld (Model: MFMR) were used that have a tip radius of ≈50 nm.

### Lamellae Preparation and Lorentz TEM

The bulk polycrystalline Heusler compound Mn_2_Rh_0.95_Ir_0.05_Sn was prepared by an arc‐melting method. Electron backscattering diffraction was performed in a TESCAN GAIA 3 (Quantax, Bruker) system to identify [001]‐oriented grains. Single‐crystalline lamellae were fabricated from single [001]‐oriented grains by a Ga^+^ ion based dual beam focused ion beam (FIB) system [FEI Nova Nanolab 600 SEM/FIB], that was operated using an accelerating voltage of 30 kV. The details of the structural characterization and fabrication can be found elsewhere.^[^ ^14^ ^]^ An aberration‐corrected high‐resolution transmission electron microscope (FEI TITAN 800‐300) was used to observe the magnetic contrast in the LTEM mode. The magnetic field was varied along the microscope axis by partially exciting the objective lens current. A double‐tilt liquid nitrogen holder was used to vary the temperature of the specimen from 100 to 300 K. The in‐plane field components were realized at the specimen surface by tilting the sample holder up to ± 32°. The defocus value for the LTEM imaging is ≈ 1.1 mm.

## Conflict of Interest

The authors declare no conflict of interest.

## Supporting information

Supporting Information

## Data Availability

The data that support the findings of this study are available from the corresponding author upon reasonable request.
