# Supplementary material for: Nanoscale Noncollinear Spin Textures in Thin Films of a D 2d Heusler Compound
Source: Adv Mater. 2021 Jul 3;33(32):2101323. doi: 10.1002/adma.202101323 (PMC11469302; doi:10.1002/adma.202101323)
Supplement: Supplementary file 1 — Supporting Information [file ADMA-33-2101323-s001.pdf]

© 2021 Wiley-VCH GmbH

ADVANCED MATERIALS

Supporting Information

for *Adv. Mater.,* DOI: 10.1002/adma.202101323

Nanoscale Noncollinear Spin Textures in Thin Films of a D_{2d} Heusler Compound

*Ankit K. Sharma, Jagannath Jena, Kumari Gaurav Rana, Anastasios Markou, Holger L. Meyerheim, Katayoon Mohseni, Abhay K. Srivastava, Ilya Kostanoskiy, Claudia Felser, and Stuart S. P. Parkin**

Supplementary information

Nanoscale non-collinear spin textures in thin films of a D_{2d} Heusler compound

Ankit K. Sharma[1], Jagannath Jena[1], K. Gaurav Rana[1], Anastasios Markou[2], Holger L. Meyerheim[1], Katayoon Mohseni[1], Abhay K. Srivastava[1], Ilya Kostanoskiy[1], Claudia Felser[2], and Stuart S. P. Parkin[1*]

[1] Max Planck Institute of Microstructure Physics, Weinberg 2, 06120 Halle, Germany

[2]Max Planck Institute for Chemical Physics of Solids, Nöthnitzer Str. 40, 01187 Dresden, Germany

Corresponding Author

Email: stuart.parkin@mpi-halle.mpg.de

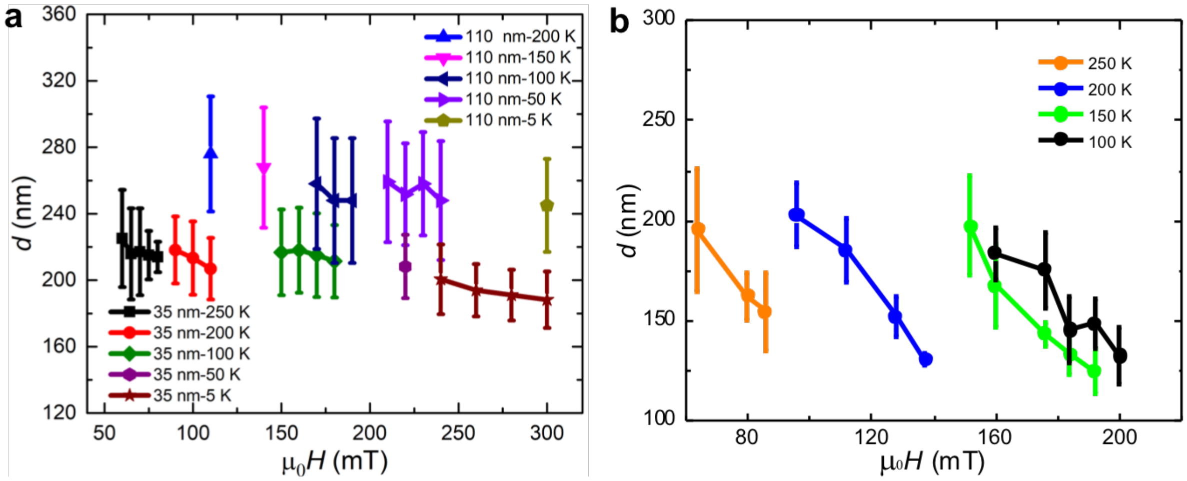

Fig. S1: **Size dependence of nanoscale objects in Mn_2RhSn thin films and $Mn_2Rh_{0.95}Ir_{0.05}Sn$ single crystalline lamella.** (a) Size dependence of nano-objects on magnetic field and temperature, for two films with thicknesses of 35 nm and 110 nm. The average size of the nano-objects varies from ~190-225 nm in the 35 nm thick film, and ~245-280 nm in the 110 nm thick film, depending on the temperature. (b) Size dependence of nano-objects on magnetic field and temperature in a 150 nm thick lamella. The size of the nano-objects varies from ~130-200 nm, depending on the temperature.

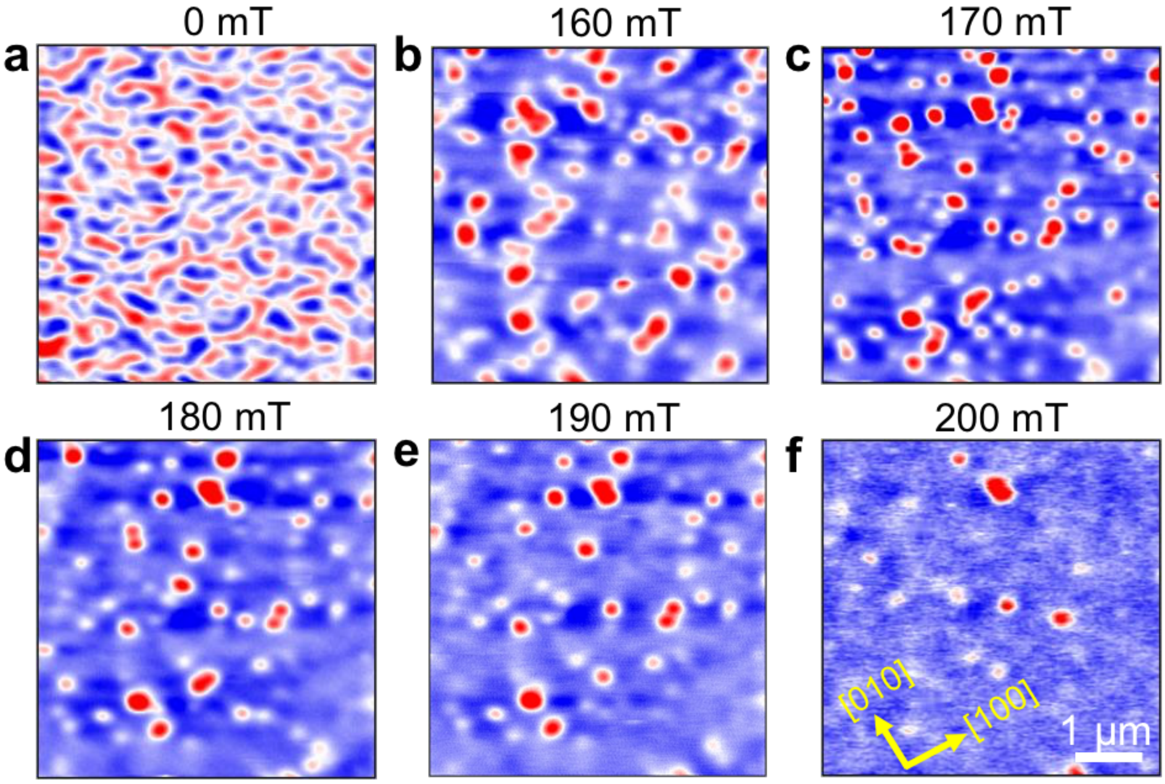

Fig. S2: **Evolution of nanoscale objects with field in a 110 nm thick Mn_2RhSn film.** (a) MFM image at 100 K and zero magnetic field. (b-f) Evolution of the MFM images as the magnetic field is increased from 160 to 200 mT. The blue and red colors correspond to up and down magnetization respectively. All images are at the same scale: a scale bar is given in (f).

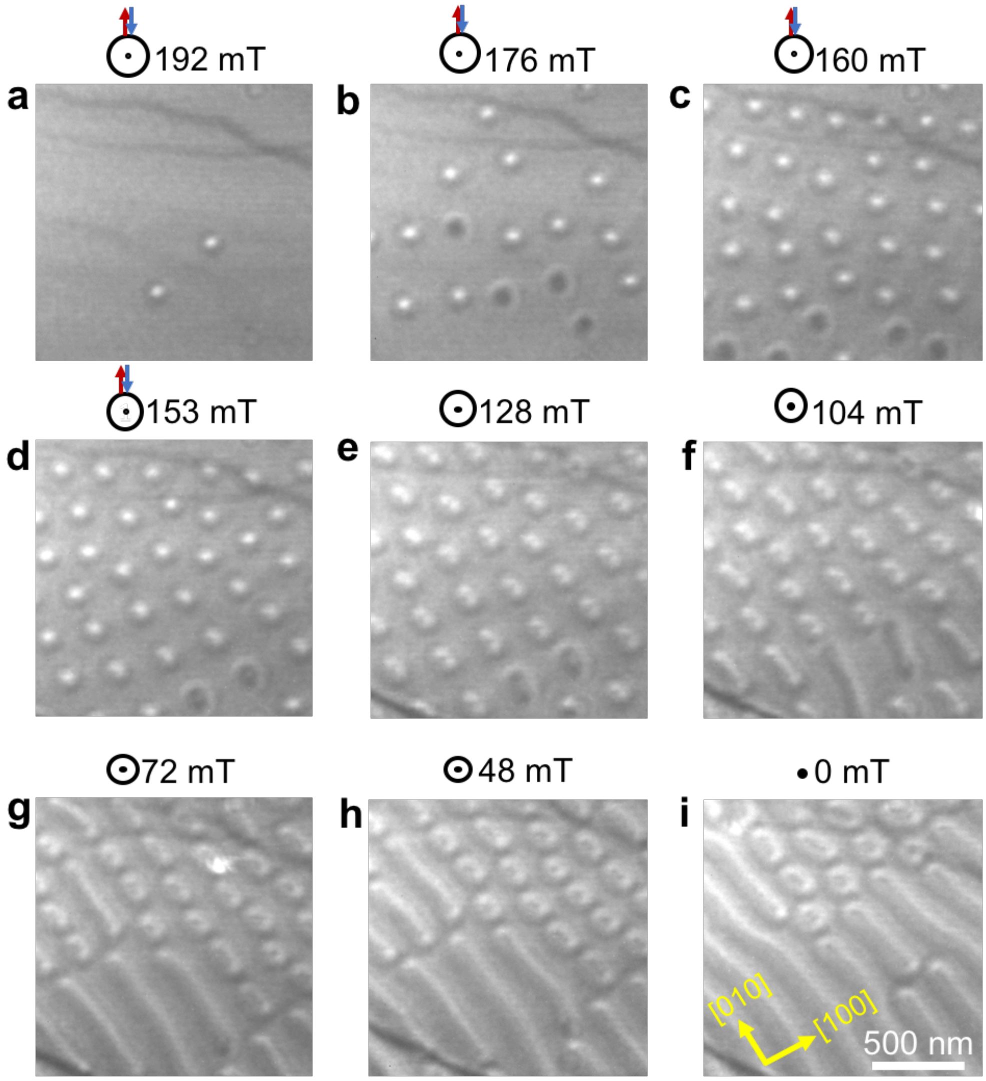

Fig. S3: Bloch skyrmions in a 150 nm thick $Mn_2Rh_{0.95}Ir_{0.05}Sn$ single crystalline lamella. The starting configuration is a field polarized state: the images shown are collected as the field is reduced. (a, b) Isolated Bloch skyrmions found at 192 mT and 176 mT, respectively. (c, d) The number density of Bloch skyrmions increases as the magnetic field is decreased. In (a-d), the red and blue arrows represent a reversible temporary tilting of the specimen that is performed to realize an in-plane field component at the specimen. (e-i) The out-of-plane field is further decreased to zero without any sample tilting. In zero field only a few nano-objects are present in the specimen. All images are at the same scale: a scale bar is given in (i).

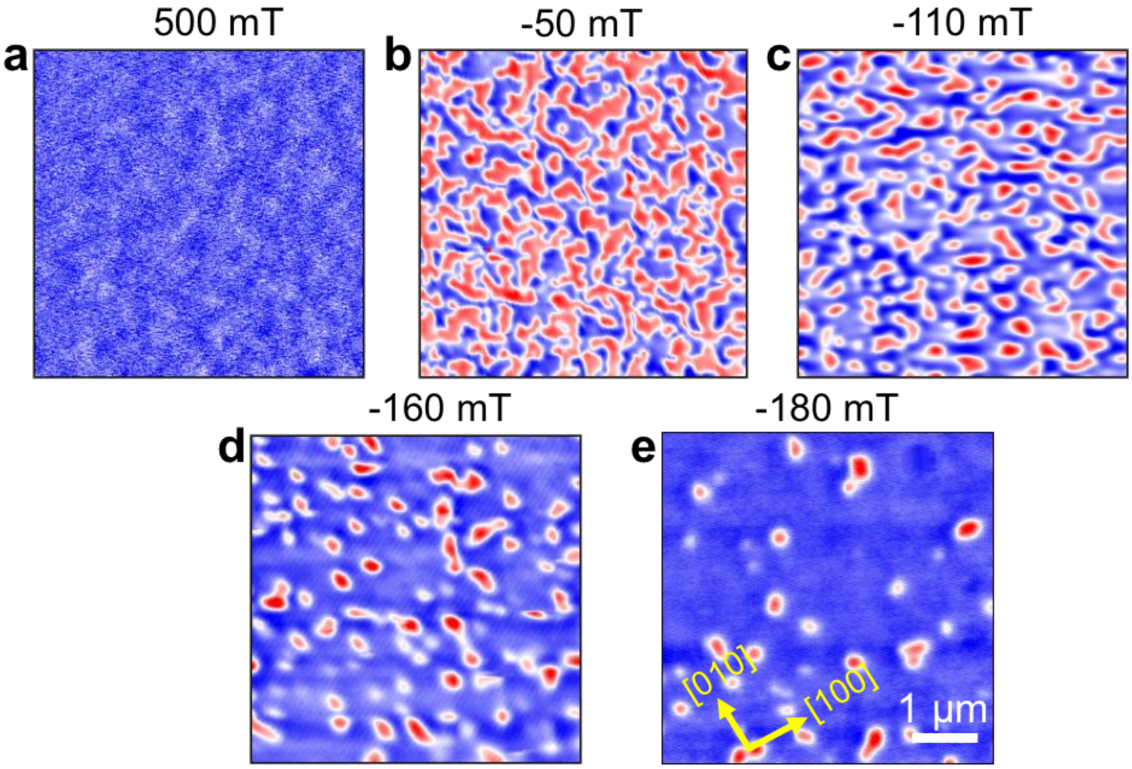

Fig. S4: **Evolution of nanoscale objects with magnetic field in a 35 nm thick Mn_2RhSn thin film**. (a-f) Magnetic field evolution of magnetic nano-objects starting from the saturated state in a large positive field, as the field is reduced through zero to negative fields. MFM images for positive field values are included in the main text in Fig. 1a-f. (a) After reaching saturation at 0.2 T the field was reduced to zero, where no contrast is observed, and the magnetization is directed along +z. When the field is reduced through zero and becomes negative, the magnetization starts switching from +z to –z, and a magnetic labyrinth phase is found, as shown in (b). The labyrinth domains start breaking up into nano-objects with both circular and elliptical shapes (c-f), which eventually transform into a saturated magnetic state for a large enough negative field (not shown). All images are at the same scale: a scale bar is given in (e).

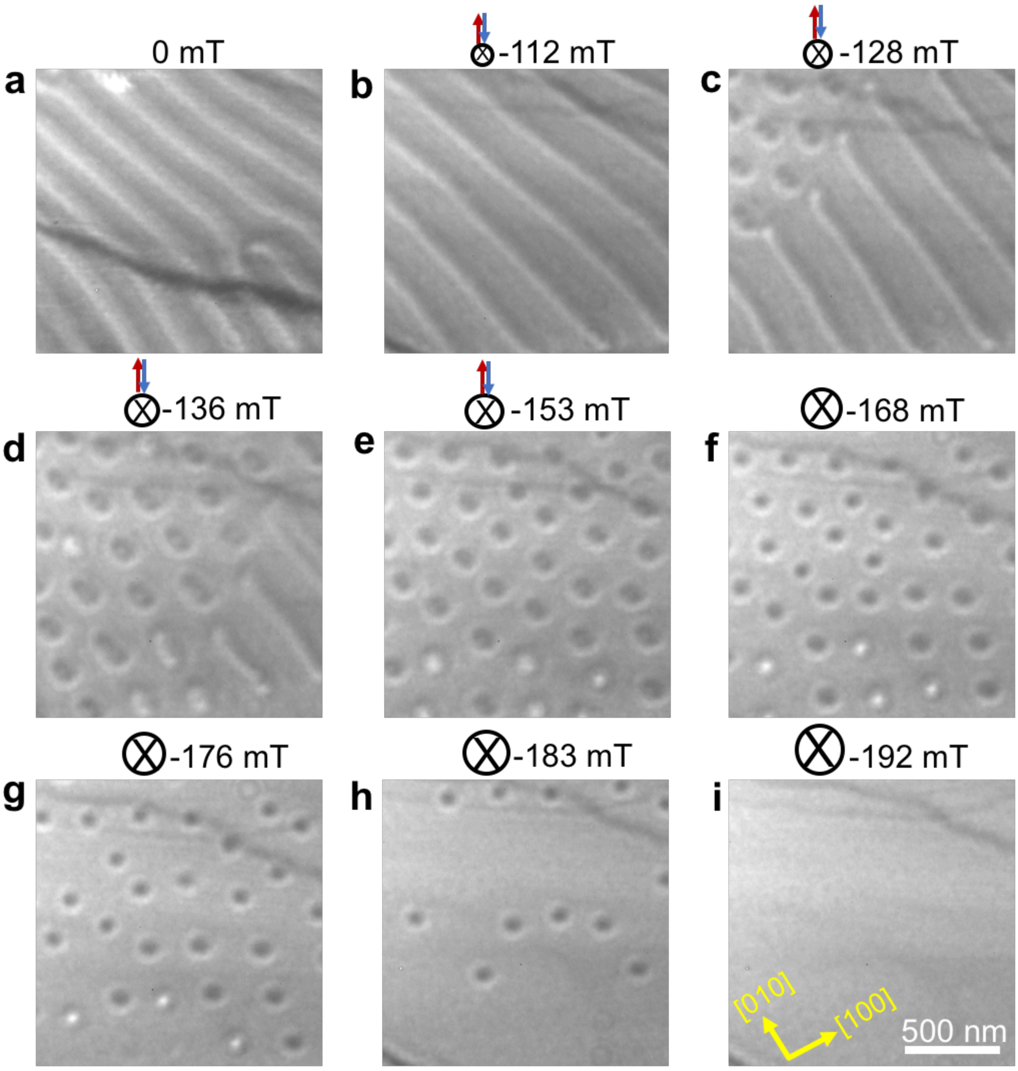

Fig. S5: Bloch skyrmions in a 150 nm thick $Mn_2Rh_{0.95}Ir_{0.05}Sn$ single crystalline lamella for negative fields. (a) The starting configuration is the helical state in zero magnetic field. (b) The helical period increases with increasing negative magnetic field. (c) Nano-objects, namely Bloch skyrmions, start to form at ~-128 mT. (d-e) The Bloch skyrmion number density increases with increasing magnitude of the negative field. (f-i) Further increasing the field, the density of Bloch skyrmions starts to decrease and at ~-192 mT a field polarized state is found. All images are at the same scale: a scale bar is given in (i).

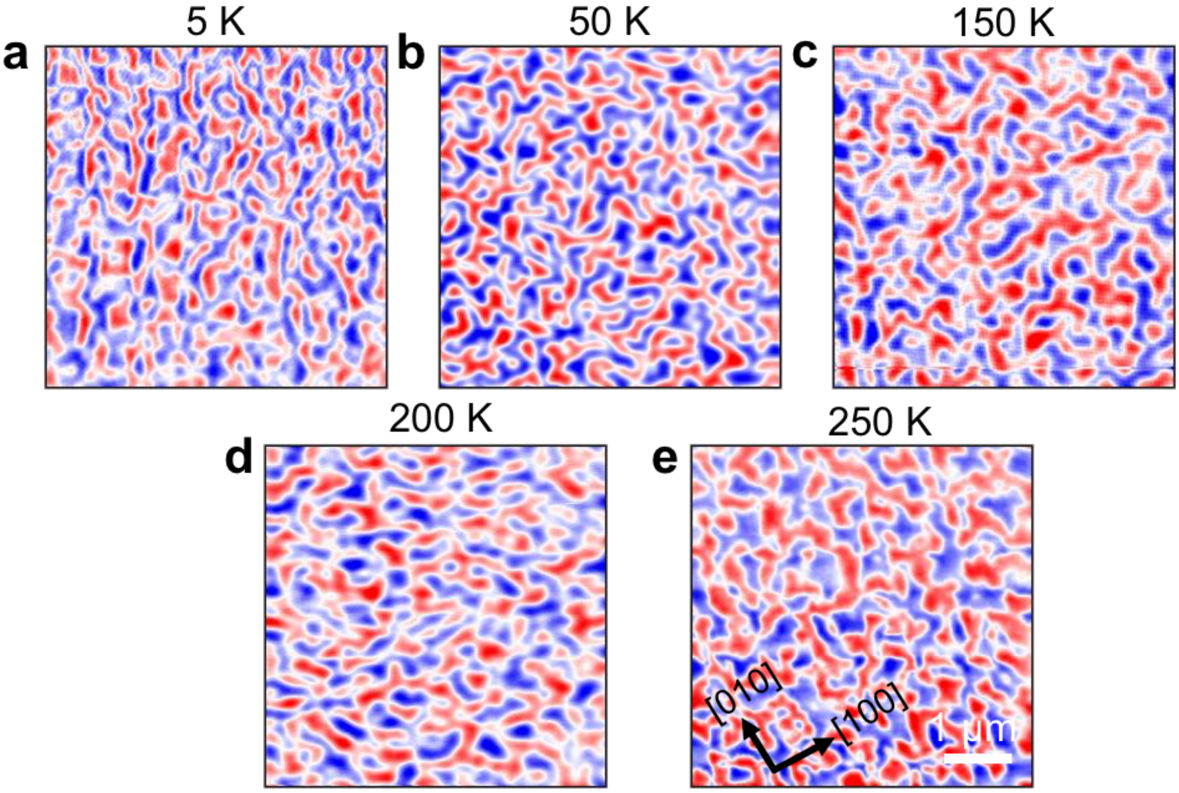

Fig. S6: **Magnetic structures in a 35 nm thick Mn_2RhSn thin film after zero field cooling from above 325 K.** MFM images of labyrinth domain phase in zero field at (a) 5 K, (b) 50 K, (c) 150 K, (d) 200 K and (e) 250 K. The film was cooled in zero field from above the Curie temperature in each case to the temperature indicated. All images are at the same scale: a scale bar is given in (e).

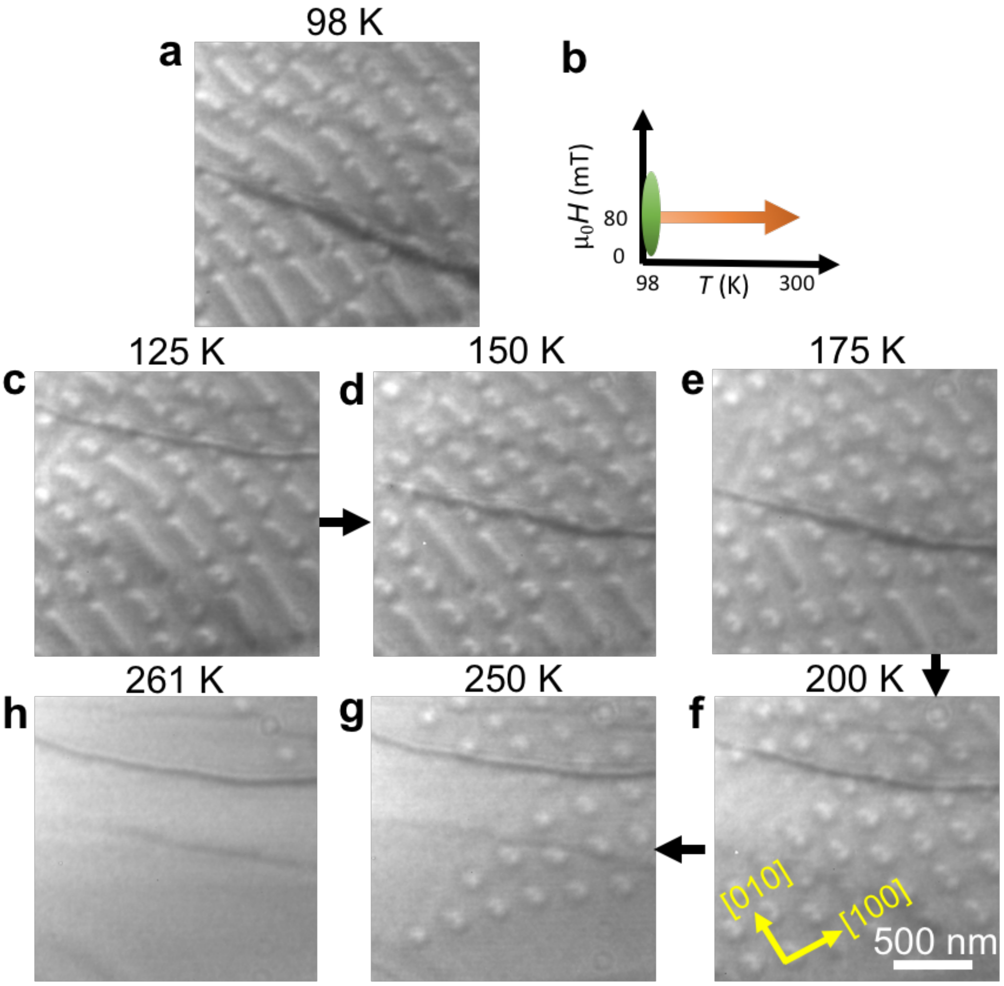

Fig. S7: Temperature dependence of Bloch skyrmions in a 150 nm thick $Mn_2Rh_{0.95}Ir_{0.05}Sn$ single crystalline lamella imaged using LTEM. (a) After stabilizing a Bloch skyrmion lattice state at a higher field and subsequently decreasing the out-of-plane field, Bloch skyrmions and short helices are found at 98 K and 80 mT. (c–h) Keeping the magnetic field constant (80 mT), the sample is heated to higher temperatures and LTEM images are taken at 125, 150, 175, 200, 250, and 261 K respectively. A schematic of the heating experiment is shown in (b). All images are at the same scale: a scale bar is given in (f).

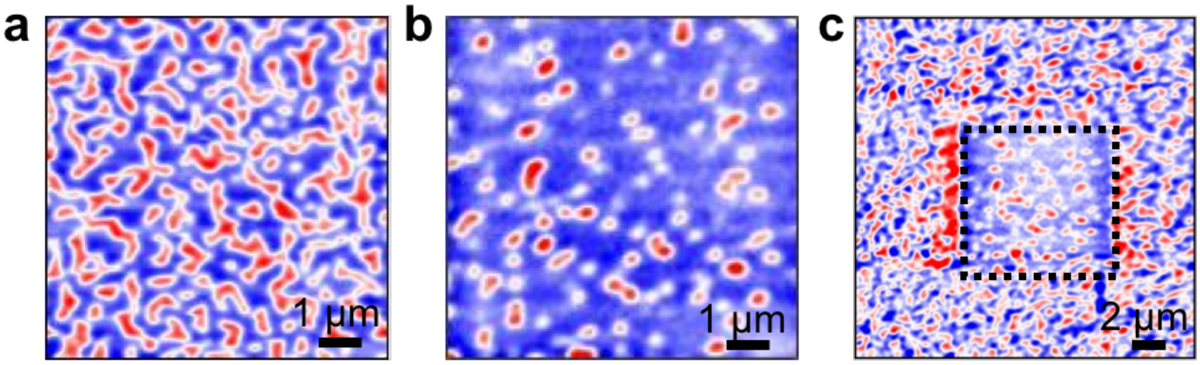

Fig. S8: **Writing nano-objects in a 35 nm thick Mn_2RhSn thin film using an MFM tip in contact mode.** (a) MFM image taken in non-contact mode at a height of 50 nm in zero field at 220 K. The blue and red colors correspond to up and down magnetization, respectively. The tip is magnetized along the +*z* direction. (b) MFM image taken in contact-mode of the same region as in (a). The magnetic field from the tip results in the elimination of the labyrinth domains and the formation of nano-objects. (c) MFM image, in non-contact mode at a height of 50 nm, of an extended area around the region shown in b. The nano-objects, which are written by the MFM tip, are marked by a dotted black square.

X-ray diffraction analysis of the 35 nm thick Mn_2RhSn film structure

A detailed investigation of the crystal structure of the 35 nm thick Mn_2RhSn film was carried out by X-ray diffraction (XRD) using an advanced Gallium-Jet X-ray source operated at 70 keV and 100 W power emitting Ga-Kα radiation (λ=1.3414 Å). The monochromatized and focused X-ray beam (horizontal and vertical beam size: 2mm x 100μm) is incident onto the sample at a constant grazing incidence angle of μ=1°. Integrated reflection intensities were collected by using a six-circle x-ray diffractometer operated in the z-axis mode [2] by rotating the sample around its surface normal (ϕ-scan) while the position of the 2-dimensional (2D) pixel detector was kept fixed at the in-plane (δ) and out-of-plane (γ) angle associated to each reflection (HKL) [2].

In total 35 reflections were measured reducing to a data set consisting of 15 symmetry independent reflections after symmetry averaging based on the 4mm point group symmetry of the diffraction pattern (out of plane momentum transfer q_z limited to $q_z>0$). The average agreement between symmetry equivalent reflections is equal to 10%. Subsequently, the observed structure factor magnitudes, $|F(HKL)_{obs}|$, were derived from the integrated intensities by multiplying with instrumental factors (Lorentz, polarization- and effective area) [3,4].

The structure refinement was carried out by least squares refinement of the calculated squared structure factor magnitudes, ($|F(HKL)_{calc}|^2$) to the observed ones ($|F(HKL)_{obs}|^2$) based on the space group (SGR) $I\bar{4}m2$ (SRG Nr. 119) by using the Program Shelx [5]. In this SGR the Mn atoms occupy the Wyckoff sites 2b at (x y z) = (0 0 $^1/_2$) and 2c (0 ½ $^1/_4$), while Sn and Rh atoms occupy Wyckoff sites 2a (0 0 0) and 2d (0 ½ $^3/_4$), respectively [6]. Owing to the high symmetry of the structure with no free positional parameters (x y z), only an overall scale factor and one common displacement parameter (ADP) [7] is allowed to vary. Based on the perfectly ordered structure we obtain a weighted (Rw) and unweighted (Ru) [8] residuum of Rw=0.29

and Ru=0.059, respectively. Although these values can be considered as not unsatisfactory, the difference Fourier synthesis exhibits a strong positive peak at the position (½ 0 ½) corresponding to the Wyckoff site 4f in SGR 119. Figure S9 shows the structure model.

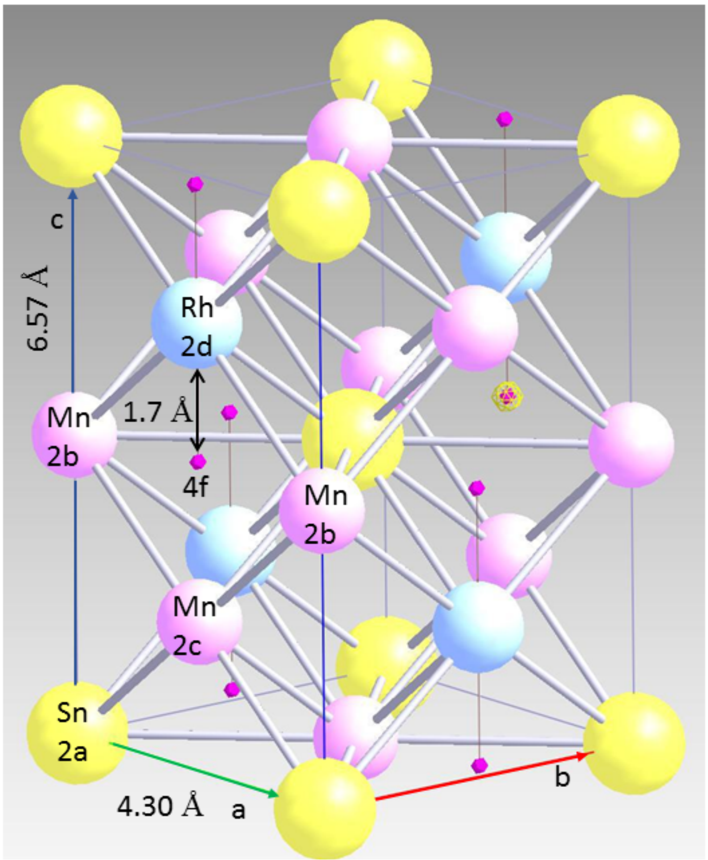

Fig. S9: XRD derived model of the 35 nm thick Mn_2RhSn film structure. Yellow, pink and blue balls represent Sn, Mn and Rh atoms, respectively. Atomic sites are labelled in accordance with their Wyckoff site in space group 119. The 4f site near (½ 0 ½) shown by small purple balls is derived from the difference Fourier synthesis of the charge density. The detailed analysis (see text) shows that this site is occupied by Mn by a concentration of about 4%, while about the same concentration of vacancies exist in the nearest neighboring sites (2c and 2d).

The strong positive difference density (see purple balls in Fig. S9) indicates that the site near (½ 0 ½) is at least partially occupied. As this site (4f in SGR119) is located by only about 1.7 Å (=c_0/4) away from neighboring Rh and Mn atoms in site 2d and 2c, the occupation of the 4f site necessarily requires the presence of vacancies in 2d and 2c to at least the same concentration. In order to quantitatively analyze the precise site occupancy of the sites involved as well as to derive the stoichiometry, systematic calculations were carried out.

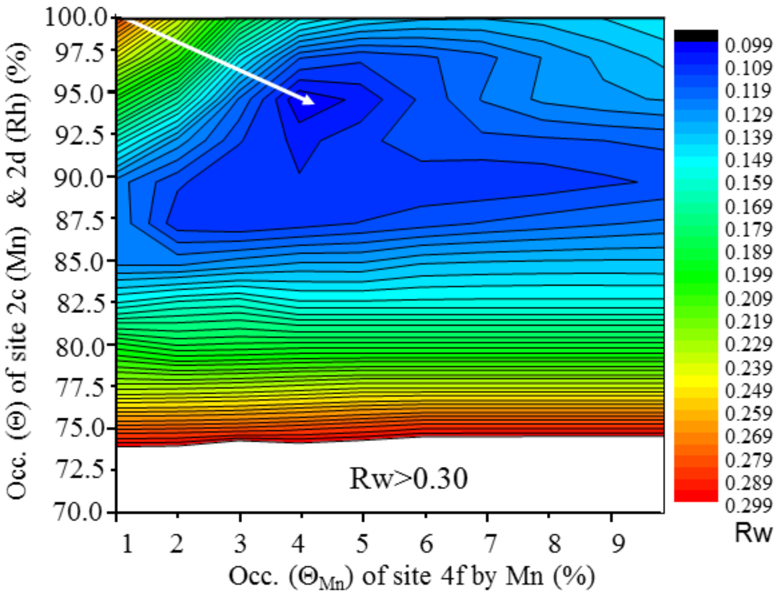

Fig. S10: Contour plot of Rw versus site occupancy (Θ) in Mn_2RhSn for site 4f versus 2d and 2c. The minimum of Rw at the end of the arrow corresponds to a concentration of Θ_{Mn}(4f) =4% and to Θ_{Mn}(2c)=Θ_{Rh}(2d)≈94%, respectively.

Fig. S10 shows the contour plot or Rw versus occupancy (Θ_{Mn}) of site 4f with Mn versus occupancy of 2d (Θ_{Rh}) and 2c (Θ_{Mn}) which are the next neighbor sites to site 4f. In the non-disordered structure the 4f site is empty, whereas 2d and 2c are completely occupied. This corresponds to Rw≈0.30 at the upper left of the plot. The fit quality is *dramatically* improved (dropping to about 1/3 of the value obtained for the perfectly ordered structure) by allowing a fraction of about 4% of the 4f site to be populated by Mn while simultaneously a vacancy

density of 6% is introduced into the 2c and 2d site. This is represented by the arrow ending at the minimum of Rw. We have also studied the possibility of other atom exchanges at other sites, but these were found to be negligible within the experimental uncertainty which we estimate to lie in the 5 % range at most. Based on this analysis we derive an average film stoichiometry which can be written as: $Mn_{2.02}$ $Rh_{0.94}$ $Sn_{1.00}$, i.e. there is a slight abundance of Mn within the film structure in relation to Rh and Sn (the concentration of the latter being normalized to 1.00).

We also examined Mn_2RhSn films for which we found no evidence of magnetic nano-objects. The XRD analysis shows that in these films chemical disorder is present which is characterized by the simultaneous statistical occupancy of Wyckoff sites 2b, 2c and 2d by Mn and Rh (2b: 88% Mn and 12% Rh, 2c: 90% Mn and 4% Rh, 2d: 90% Mn and 5%Rh). This chemical disorder modifies the stoichiometry of the film to $Mn_{1.90}$ $Rh_{1.064}$ $Sn_{1.00}$, i.e., it contains less Mn and more Rh than those films where less chemical disorder is observed and which show magnetic nano-objects. Thus, we conclude that films which show no nano-objects are more chemically disordered than the films presented in this manuscript.

References:

[1] T. Ma, A. K. Sharma, R. Saha, A. K. Srivastava, P. Werner, P. Vir, V. Kumar, C. Felser, S. S. P. Parkin, *Adv. Mater.* **2020**, 32, 2002043.

[2] E. Vlieg, and M. Lohmeier, *J. Appl. Cryst.* **1993**, *26*, 706.

[3] C. Schamper, H. L. Meyerheim, and W. W. Moritz, *J. Appl. Cryst*. **1993**, *26*, 687.

[4] E. Vlieg, *J. Appl. Cryst*. **1997**, 3*0*, 532.

[5] G. M. Sheldrick, *Acta. Cryst* **2015**, *C71*, 3.

[6] International Tables for Crystallography. Volume A, Space-Group Symmetry. Dordrecht; London: Published for the International Union of Crystallography by Kluwer Academic Publishers, **2002**.

[7] W. F. Kuhs, *Acta Cryst.* **1992**, *A48*, 80.

[8] The unweighted (Ru) and the weighted residuum are given by:

$Ru = \frac{\sum ||F_o| - |F_C||}{\sum |F_o|}$ $Rw = \sqrt{\left\{\frac{\sum [w(F_o^2 - F_c^2)^2]}{\sum [wF_o^2]^2}\right\}}$, where for short Fo and Fc are the observed and calculated stricture factor magnitudes and the summation runs over all reflections (HKL).
